# Characterization of RBP9 and RBP10, two developmentally regulated RNA-binding proteins in *Trypanosoma brucei*

**DOI:** 10.1098/rsob.160159

**Published:** 2017-04-05

**Authors:** Luis Miguel De Pablos, Steve Kelly, Janaina de Freitas Nascimento, Jack Sunter, Mark Carrington

**Affiliations:** 1 Department of Biochemistry, University of Cambridge, Cambridge CB2 1QW, UK; 2 Centre for Immunology and Infection (CII). Biology Dept., University of York, York YO10 5DD, UK; 3 Department of Plant Sciences, University of Oxford, South Parks Road, Oxford OX1 3RB, UK; 4 Sir William Dunn School of Pathology, University of Oxford, Oxford OX1 3RE, UK

**Keywords:** RNA, gene expression, differentiation, kinetoplastid, biotin identification

## Abstract

The fate of an mRNA is determined by its interaction with proteins and small RNAs within dynamic complexes called ribonucleoprotein complexes (mRNPs). In *Trypanosoma brucei* and related kinetoplastids, responses to internal and external signals are mainly mediated by post-transcriptional processes. Here, we used proximity-dependent biotin identification (BioID) combined with RNA-seq to investigate the changes resulting from ectopic expression of RBP10 and RBP9, two developmentally regulated RNA-binding proteins (RBPs). Both RBPs have reduced expression in insect procyclic forms (PCFs) compared with bloodstream forms (BSFs). Upon overexpression in PCFs, both proteins were recruited to cytoplasmic foci, co-localizing with the processing body marker SCD6. Further, both RBPs altered the transcriptome from a PCF- to a BSF-like pattern. Notably, upon expression of BirA*-RBP9 and BirA*-RBP10, BioID yielded more than 200 high confidence protein interactors (more than 10-fold enriched); 45 (RBP9) and 31 (RBP10) were directly related to mRNA metabolism. This study validates the use of BioID for investigating mRNP components but also illustrates the complexity of mRNP function.

## 1. Introduction

The fate of an mRNA is determined by its interaction with proteins and small RNAs, which control its stability, translatability and localization within the cell. These proteins are either RNA-binding (RBPs) or part of a complex that contains RBPs. The complement of proteins bound to any mRNA is dynamic and alters on export from the nucleus to the cytoplasm and in response to changes, for example external stress [1–3], internal cell cycle rhythms [4] or circadian rhythms [5,6]. In this context, protein targeting and localization of the mRNPs play an important role in the regulation of the rates of translation and ensure that the translation of the mRNA responds to changing conditions [7–9].

*Trypanosoma brucei* has a complex life cycle with 10 or more different developmental forms in the insect vector and the mammalian host. Uniform populations of two forms of the life cycle can be routinely cultured *in vitro*: the bloodstream form (BSF) representative of the stage found in the blood and tissue fluids of mammalian hosts, and the procyclic form (PCF), one of the stages present in the gut of the tsetse fly vector. Adaptation to the two distinct environments has resulted in major differences in morphology, surface composition and biochemical pathways, all driven by stage-specific gene expression [10–12]. The *T. brucei* genome is organized into long polycistronic units which are constitutively transcribed [13–15] and co-transcriptionally processed into monocistronic mRNA molecules by a *trans*-splicing reaction that involves the addition of a 39 nt spliced leader sequence and the polyadenylation of the 3′-end [16–18]. The lack of canonical RNA polymerase II promoters and the polycistronic transcription unit (PTU) organization means that regulation through selective transcription of individual genes does not operate [13,19,20].

During the trypanosome life cycle, transitions between developmental forms occur in response to external cues such as a change in temperature and chemical milieu [10,21,22]. The expression of the vast majority of mRNAs does not change: in *T. brucei* one analysis indicated that 5.6% of mRNAs are differentially expressed between BSFs and PCFs [23]. In addition, ribosomal profiling revealed that there are specific subsets of mRNAs that are differentially translated between BSFs and PCFs including proteins related to energy metabolism, surface proteins, phosphatases and RBPs [24]. These subsets of mRNAs also show developmental differences in their half-life [25], with components of the 5′ to 3′ exonucleolytic degradation pathway including DHH1 and XRNA being involved in the turnover of developmentally regulated mRNAs [25,26].

Several studies have demonstrated the important role of RBPs as *trans*-acting factors in the control of developmental transitions [27]. The best example of this is the overexpression of RBP6 in PCFs that leads to the progression through a series of different developmental forms found in the tsetse fly [28]. Other examples include: overexpression of ALBA 3/4 impairing the normal differentiation that occurs in the tsetse fly proventriculus [29]; ZFP3 stabilizing developmentally regulated mRNAs [30,31]; and overexpression of RBP10 in PCFs inducing many BSF transcripts [32].

Some complexes involved in mRNA metabolism have been characterized in trypanosomes: the spliceosome responsible for the *trans*-splicing of transcripts [33,34]; the exosome [35]; and the CAF1–NOT complex involved in deadenylation and decay of mRNA [36]. However, little is known about the composition of other specialized mRNPs and how they change in response to environmental cues. The full composition of many mRNPs is currently at the edge of, or beyond, available technology. The problem has three main components: the low copy number of most mRNAs; difficulty in purifying individual mRNPs; and the dynamic nature of interactions leading to losses during purification.

In this study, we report the combination of biotin identification (BioID) and RNA-seq as an approach to defining the composition of mRNPs implicated in developmental regulation in *T. brucei*. RBP9 and RBP10 were analysed following overexpression in PCFs: each resulted in changes to developmentally regulated mRNAs and favoured expression of BSF mRNAs. Proximity biotinylation identified overlapping sets of proteins that favoured proteins involved in mRNA metabolism.

## 2. Material and methods

### 2.1. Cell lines and vectors

PCFs of *Trypanosoma brucei* Lister 427 (a kind gift of Keith Gull, University of Oxford) were modified by integration of pSPR2, to constitutively express Tet repressor protein [37], and used for all experiments. Trypanosomes were cultured at 27°C in SDM-79 media supplemented with 10% fetal calf serum [38].

Overexpression was from tetracycline-inducible transgenes, driven by an EP procyclin promoter, integrated into the rRNA locus [37]. Transgenic parasites were generated using standard procedures [39], and transfectants were selected by adding the appropriate selection marker and clonal populations obtained by limiting dilution.

For expression of the wild-type proteins, the open reading frames (ORFs) of RBP10 and RBP9 were cloned into the vector p3383 [37]. A similar approach using the vector p3888 [37] produced tetracycline-inducible transgenes encoding either eYFP-RBP9 or eYFP-RBP10. For BioID assays, the myc-BirA* module from pLew100mycBirA* (a kind gift of Brooke Morriswood, Wuerzburg University) [40] was cloned into p3383 to make p4521, a vector for tetracycline-inducible expression of transgenes tagged at the N-terminus with BirA*. The RBP10, RBP9 and GFP ORFs were individually cloned into p4521 and used to transfect trypanosomes. After integration, these constructs resulted in cell lines containing tetracycline-inducible transgenes encoding myc-BirA*-RBP9, myc-BirA*-RBP10 or myc-BirA*-GFP. As previously, mChFP-SCD6 and mChFP-DHH1 were expressed by modifying the endogenous locus [41].

### 2.2. RNA extraction, northern blotting and RNA-seq

RNA for northern blots and RNA-seq was prepared using Qiagen RNAeasy kits at a series of time points after induction of transgene expression.

Northern blots were performed as previously described [42]. RNA probes were full length ORFs of mitochondrial glutamate dehydrogenase (Tb927.9.5900), mitochondrial malate dehydrogenase (Tb927.10.2560), glycosomal aldolase (Tb927.10.5620) and metacaspase MCA4 (Tb927.10.2440). Ribosomal RNA was used to measure loading.

For RNA-seq experiments, cDNA libraries were made and sequenced at the Beijing Genomics Institute (Shenzhen, China). Polyadenylated RNA was purified from total RNA, converted to cDNA using random hexamer primers, sheared and size selected for fragments approximately 200 bp in length using the Illumina TruSeq RNA Sample Preparation Kit v2. Sequencing was performed on an Illumina Hiseq 2000 (Illumina, CA) yielding 90 base paired end reads. RNA-seq reads of the resulting library was used for the determination of transcript abundances, using approximately 10 million reads per sample.

Raw sequence reads were quality-trimmed using Trimmomatic [43]. This was done to remove low-quality bases and read-pairs as well as contaminating adaptor sequences prior to abundance estimation. Reads were searched for all common Illumina adaptors (the default option) and the settings used for read processing by Trimmomatic were LEADING:10 TRAILING:10 SLIDINGWINDOW:5:15 MINLEN:50. Trimmed read-pairs were aligned to the complete set of transcripts from version 6 of the *T. brucei* TREU 927 genome [44] using Bowtie 2 [45] and transcript abundances estimated from these mapped reads using RSEM [46].

### 2.3. Live cell imaging

Trypanosomes expressing fluorescent fusion proteins were visualized by live cell microscopy. Cells from 1 ml culture were centrifuged at 10 000 r.p.m. for 1 min, washed once with SDM-79 without serum and resuspended in 50 µl of SDM without serum. DNA was stained with Hoechst H33342 and images were taken within the next 10 min using a Zeiss Axioimager M1. Images were recorded using the Axiovision software (Zeiss) and then imported into Adobe Photoshop. A minimum of 100 cells from randomly selected fields was used for P-body counts.

### 2.4. Flow cytometry

Parasites with or without doxycycline (DOX) induction were analysed using a BD FACScan (BD Biosciences). The histograms of the different cell lines were generated using Summit software (Beckman Coulter, Inc).

### 2.5. Polysome fractionation and western blots

Polysome analysis was performed as previously described [47]; western blots were performed using standard protocols. Streptavidin-POD (Jackson Immunoresearch) was diluted 1/1000, anti-myc 1/1000, anti-BiP 1/1 0000, anti-P0 1/2500 and anti-eIF4A1 used as loading control 1/5000 [48].

### 2.6. Affinity capture of biotinylated proteins

All three inducible cell lines ectopically expressing BirA*-RBP10, BirA*-RBP9 and BirA*-GFP fusion proteins were grown in medium supplemented with 50 µM biotin and then induced with 1 µg ml^−1^ DOX. For small-scale experiments 5 × 10^8^ cells were used, and 5 × 10^9^ cells for large-scale experiments. The cells were harvested at 1800*g* for 10 min and washed twice with v-PBS (PBS pH 7.6, plus 40 mM sucrose, 10 mM glucose). Then, the cell pellets were incubated on ice with Lysis Buffer (20 mM Tris–HCl pH 7.5, 150 mM NaCl, 1% *n*-octyl-glucoside and 1× Complete protease inhibitor without EDTA (Roche)). After the incubation, the lysates were centrifuged at 16 000*g* for 3 min at 4°C and the supernatants incubated with 500 µl of streptavidin magnetic beads (Dynabeads, MyOne Streptavidin C1; Invitrogen) for 30 min with gentle agitation. The beads were collected using a magnet and washed twice in PBS, 0.1% SDS, and twice in PBS for a total of four washes. Finally, bound biotinylated proteins were eluted by incubation in 50 µl SDS-PAGE loading buffer for 5 min at 98°C.

### 2.7. Mass spectrometry analysis

Purified proteins from the two independent biological replicates per sample were separated on a NuPAGE bis-tris 4–12% gradient polyacrylamide gel (Invitrogen) under reducing conditions. The sample lane was divided into five slices that were excised from the Coomassie stained gel, destained, then subjected to tryptic digest and reductive alkylation. Liquid chromatography tandem mass spectrometry (LC-MS/MS) was performed by the Proteomic Facility at the University of Dundee. The five fractions obtained from SDS-PAGE were subjected to LC-MS/MS on an UltiMate 3000 RSLCnano System (Thermo Scientific) coupled to an LTQ OrbiTrap Velos Pro (Thermo Scientific), and mass spectra were analysed using MaxQuant v. 1.5 [49] searching the *T. brucei* TREU927 annotated protein database (release 8.1) from TriTrypDB (http://tritrypdb.org/tritrypdb/). Minimum peptide length was set at six amino acids, isoleucine and leucine were considered indistinguishable and false discovery rates of 0.01 were calculated at the levels of peptides, proteins and modification sites based on the number of hits against the reversed sequence database.

Normalized spectral index quantitation (SINQ) was used for quantitation [50]. Enrichments were calculated by comparing SINQ values for each protein identified in BirA*-RBP9 and BirA*-RBP10 samples with BirA*-GFP SINQ values. An arbitrary background value (Spectral Index (MIC SIn) = 5 × 10^−9^) was assigned to unique hits found in BirA*-RBP9 and BirA*-RBP10 samples.

## 3. Results

### 3.1. Selection of RBPs for overexpression in PCFs and experimental strategy

**Figure 1.**
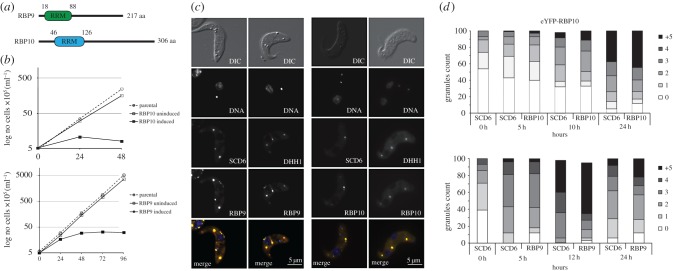
Overexpression of RBP9 and RBP10 in PCFs. (*a*) Schematic of RBP9 and RBP10 proteins. RRM, RNA recognition motif. (*b*). Cumulative growth curve of parental (Lister 427 pSmOx), induced (+DOX) and uninduced (−DOX) cell lines for RBP9 (Lister 427 pSmOx p4632) and RBP10 (Lister 427 pSmOx p4509). (*c*) Co-localization of eYFP-RBP9 and eYFP-RBP10 proteins with P-body markers. Cells 12 h post-induction with endogenous mChFP-SCD6 (Lister 427 pSmOx p4475 p3626 and Lister 427 pSmOx p4423 p3626) and mChFP-DHH1 (Lister 427 pSmOx p4475 p3625 and Lister 427 pSmOx p4423 p3625). (*d*) Granule formation kinetics over a series of time points (0–24 h). One hundred cells selected at random from images were analysed, and the mChFP-SCD6, eYFP-RBP9 and eYFP-RBP10-associated granules were counted for each of the cells.

Transcriptome data [23] were screened for RBP mRNAs expressed at a higher level in BSFs than in PCFs and two RNA-binding proteins, RBP9 (Tb927.11.12120) and RBP10 (Tb927.8.2780), were chosen for further study (table 1). Both RBP9 and RBP10 contain a single RRM domain but outside this domain have no obvious identity to characterized genes in organisms outside the kinetoplastids (figure 1*a*). Inducible overexpression in PCFs was selected as an experimental approach as this has previously been more informative on function of RNA-binding proteins than RNAi-mediated knock down [28,32]. Cell lines containing tetracycline-inducible transgenes for each protein of interest were then used to determine both changes in the transcriptome and the proteins interacting with each RBP after induction.

**Table 1.** Developmentally regulated RBPs derived from transcriptome data [23].

identifier	product	BSF/PCF	references
Tb927.11.12120	RBP9	7	[32]
Tb927.8.2780	RBP10	4.1	
Tb927.6.3480	DRBD5	4.1	[28]
Tb927.3.2930	RBP6	2.3	
Tb927.7.3730	RBP26	2.3	[27]
Tb927.7.4730	PUF5	2.1	

### 3.2. Overexpression of RBP9 and RBP10 cause an arrest in proliferation

In initial experiments, the effect of overexpression of RBP9 or eYFP-RBP9 on cell proliferation was determined. In both cases, proliferation slowed at approximately 12 h post-induction and stopped by 24 h (figure 1*b*; electronic supplementary material, figure S1). When RBP10 or eYFP-RBP10 was overexpressed, proliferation slowed at approximately 5 h and ceased by 10 h (figure 1*b*; electronic supplementary material, figure S1). The presence of the N-terminal eYFP tag had no obvious effect on the proliferation phenotype. The relative expression of the eYFP-RBPs was determined using flow cytometry to measure eYFP fluorescence: both eYFP-RBPs reached similar levels (electronic supplementary material, figure S2).

Next, the subcellular localizations of eYFP-RBP9 and eYFP-RBP10 were determined after induction. Both proteins were distributed in the cytosol and also in foci, with an irregular distribution of those granules throughout the cytoplasm (figure 1*c*). Both RBP9 and RBP10 co-localized with mChFP-SCD6, a marker for mRNA processing bodies (P-bodies; figure 1*c*). Over a time course after induction, the number of visible P-bodies per cell increased. For eYFP-RBP9, an increase in P-body number occurred as proliferation slowed at 5 h. The increase in number on eYFP-RBP10 expression was less pronounced and was greatest after proliferation ceased and was still increasing at 24 h. By contrast, the maximum number of P-bodies occurred at 12 h for eYFP-RBP9. This increase in foci was visible for both eYFP-RBPs and mChFP-SCD6 (figure 1*d*), suggesting a real increase in P-body number. This increase in P-body number was expected as it usually occurs as cells reduce or cease proliferation [47].

### 3.3. RBP9 or RBP10 overexpression results in a transcriptome with BSF features

**Figure 2.**
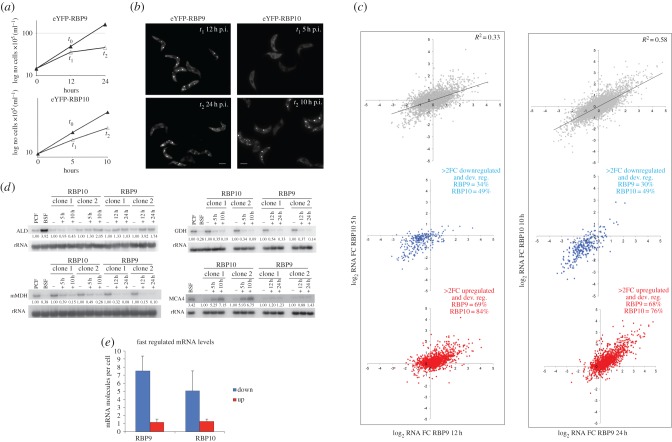
The effect of RBP9 and RBP10 overexpression on the transcriptome in PCFs. (*a*) Growth curve and time points selected for eYFP-RBP9 (Lister 427 pSmOx p4475) (*t*_0_: –DOX; *t*_1_ : 12 h +DOX; *t*_2_ : 24 h +DOX) and eYFP-RBP10 (Lister 427 pSmOx p4423)( *t*_0_ : 5 h–DOX; *t*_1_ : 5 h +DOX; *t*_2_ : 10 h +DOX). (*b*) Cell imaging of eYFP-RBP9 (Lister 427 pSmOx p4475) and eYFP-RBP10 (Lister 427 pSmOx p4423) cells at *t*_1_ and *t*_2_. (*c*) Scatter plot of Log2 fold change (FC) values for eYFP-RBP9 versus eYFP10-RBP10 at *t*_0_, *t*_1_ and *t*_2_. Red dots represent mRNAs normally upregulated in BSFs. Blue dots represent mRNAs normally downregulated in BSFs. (*d*) Northern blots validation of mRNAs affected by RBP overexpression in PCF WT, BSF WT, RBP9 at *t*_0_, *t*_1_ and *t*_2_ and RBP10 at *t*_0_, *t*_1_, *t*_2_. rRNA was used as loading control. ALD: fructose-bisphosphate aldolase, glycosomal (Tb927.10.5620); mMDH: mitochondrial malate dehydrogenase (Tb927.9.5900); GDH: glutamate dehydrogenase (Tb927.9.5900); MCA4: metacaspase MCA4 (Tb927.10.2440). (*e*) mRNA molecules per cell at *t*_0_ for transcripts affected immediately by RBP overexpression (more than twofold change (2FC) at *t*_1_). Blue bars represent transcripts downregulated at *t*_1_. Red bars represent transcripts upregulated at *t*_1_.

To screen for the effects of the induction of eYFP-RBP9 and eYFP-RBP10 on mRNAs in PCFs, a transcriptome analysis over a series of time points was performed. The time points were chosen to coincide with the time at which proliferation slowed and twice that length of time, as an attempt to distinguish primary and secondary events. The following time points were therefore used for RBP9: *t*_0_ = uninduced, *t*_1_ = 12 h, *t*_2_ = 24 h; and for RPB10: *t*_0_ = uninduced, *t*_1_ = 5 h, *t*_2_ = 10 h (figure 2*a*,*b*).

Data for the following were removed prior to analysis of the transcriptome: (i) multigene families as allocation of reads to individual genes was not possible; (ii) gene families where the copy number is unknown, for example ESAGs; and (iii) genes with an expression level below 20 fragments per kilobase per million mapped reads (FPKM). This left 7624 mRNAs for analysis.

The effect of transgene induction on the overall transcriptome was analysed initially by comparing mRNA levels expressed as FPKM reads (FPKM) (electronic supplementary material, table S1 and figure S3). First, the two uninduced cell lines were compared and there was a good correlation (*R*^2^ = 0.928). Overexpression of eYFP-RBP9 had altered the transcriptome by the time cell proliferation slowed (*R*^2^ = 0.834, *t*_1_ versus *t*_0_), and this change increased (*R*^2^ = 0.754, *t*_2_ versus *t*_0_). By contrast, overexpression of eYFP-RBP10 had little effect on the overall transcriptome at the time point when proliferation had slowed (*R*^2^ = 0.933, *t*_1_ versus *t*_0_) although a change was found at *t*_2_ (*R*^2^ = 0.834, *t*_2_ versus *t*_0_).

Next mRNAs with a twofold or greater change with respect to *t*_0_ after induction were identified. For eYFP-RBP9, there were 352 mRNAs upregulated and 357 downregulated at *t*_1_ (9.2% of the analysed transcriptome), increasing to 554 upregulated and 533 downregulated genes at *t*_2_ (14.2% of the analysed transcriptome; table 2; electronic supplementary material, table S2). The mRNAs with an altered expression level contained a substantial number of developmentally regulated mRNAs (table 2). For this analysis, developmentally regulated mRNAs were identified by comparing transcript levels determined by RNA-seq in wild-type BSF and PCF cells from isogenic cell lines and using a twofold or greater change in an mRNA between life cycle stages as the criterion for developmental regulation (EBI array express accession number E-MTAB-3335). Over half the transcripts altered by eYFP-RBP9 expression were normally developmentally regulated: those upregulated by RBP9 expression were upregulated in BSFs and those downregulated by RBP9 expression were downregulated in BSFs (figure 2*c* and table 2). Of the mRNAs affected by RBP9 expression, the fraction that is normally developmentally regulated decreased between *t*_1_ and *t*_2_, especially for the downregulated fraction. This decrease might be the consequence of secondary effects of proliferation arrest at *t*_2_ (figure 2*a* and table 2). The list of regulated transcripts was compared with mitochondrial and glycosomal protein datasets [40,41] as energy metabolism is one of the major differences between PCF and BSF cells. At *t*_2_, overexpression of eYFP-RBP9 altered the expression of 245 mRNAs related to the mitochondrion and 57 to the glycosome, representing 54.9% of all of mRNAs with altered expression (table 2; electronic supplementary material, table S2).

**Table 2.** Numbers of mRNAs with twofold or greater change in expression after transgene expression.

	RBP9				RBP10			
	*t*_1_ (12 h)		*t*_2_ (24 h)		*t*_1_ (5 h)		*t*_2_ (10 h)	
	down	up	down	up	down	up	down	up
≥2-fold change	352	357	554	533	127	135	302	360
mitochondrial	143	24	208	37	48	10	123	22
glycosomal	30	11	42	15	13	8	17	10
% of total	49	10	45	10	48	13	47	9
dev. reg.	118	248	168	362	62	113	124	275
% of total	34	69	30	68	49	84	41	76

On analysing the mRNAs altered more than twofold by eYFP-RBP10 expression, a total of 135 were upregulated and 127 downregulated at *t*_1_ (3.4% of the analysed transcriptome), increasing to 360 upregulated and 302 downregulated genes at *t*_2_ (8.6% of the analysed transcriptome; table 2; electronic supplementary material, table S3). As with RBP9, many of the regulated transcripts were also developmentally regulated (66% and 58% at *t*_1_ and *t*_2_, respectively), representing 84% and 76% of the upregulated genes at *t*_1_ and *t*_2_, respectively (figure 2*c* and table 2; electronic supplementary material, table S3). Again, among the mRNAs affected by RBP10 expression, there was a decrease in the fraction that was also developmentally regulated between *t*_1_ and *t*_2_ (table 2). In the case of eYFP-RBP10 overexpression, a comparison of affected mRNAs with mitochondrial and glycosomal proteomes showed alterations in 145 mitochondrial and 27 glycosome mRNAs at *t*_2_, representing 55.5% of the total downregulated RNAs (table 2; electronic supplementary material, table S3). The alterations to the transcriptome after eYFP-RBP10 expression described here are in good agreement with a previous microarray of PCF cell lines ectopically expressing RBP10 (*R*^2^ = 0.72) [32], confirming similar expression patterns in both PCF cell lines after induction of RBP10 (electronic supplementary material, figure S4).

The transcriptome analysis was not designed to provide a comprehensive measure of all the changes to the transcriptomes; although there is some internal duplication, only single cell lines for each transgene were used. The aim was to identify some mRNAs with obvious and large changes. To validate this approach, some of the individual mRNAs that changed in the RNA-seq data were analysed by northern blotting, using the same cell lines used for the RNA-seq analysis (clone 2) and a further independent clone (clone 1; figure 2*d*). Three mRNAs altered by both RBP9 and RBP10 overexpression were used: mitochondrial glutamate dehydrogenase (mGDH Tb927.9.5900), mitochondrial malate dehydrogenase (mMDH Tb927.10.2560) and glycosomal aldolase (ALD Tb927.10.5620) (figure 2*c*). In the case of mGDH and mMDH, expression of the mRNAs was reduced to BSF levels by *t*_1_. By contrast, the levels of aldolase mRNA increased towards, but did not reach, BSF levels by *t*_1_ in clone 2; this increase did not occur in clone 1 for reasons we do not understand. These results largely corroborated the changes observed by RNA-Seq.

An analysis to determine the average copy number of mRNAs that were up- or downregulated after transgene expression was performed as previously described [51]. mRNA copy numbers were calculated from FPKM values using PGKB mRNA as a reference [51]. Transcripts were separated into fast (mRNAs with a twofold or greater change at *t*_1_) and slow (mRNAs with a twofold or greater change only at *t*_2_) regulated and the mean copy number for each group calculated (figure 2*e*; electronic supplementary material, table S4). The analysis showed that many mRNAs that were upregulated after transgene induction had low initial mRNA copy numbers for both fast- and slow-regulated transcripts (RBP9: fast, 1.2/cell and slow, 1.6/cell; RBP10: fast, 1.3/cell and slow, 1.1/cell), whereas downregulated mRNA had a higher copy number for both fast- and slow-regulated transcripts (RBP9: fast, 7.5/cell and slow, 6.8/cell; RBP10, fast, 5.1/cell and slow, 8/cell). These numbers emphasize the contribution of developmentally regulated mRNAs to the transcriptome changes after either RBP9 or RBP10 overexpression.

The expression of many mRNAs was altered in a similar manner by both RBP9 and RBP10 overexpression. This was quantified by plotting the fold change in mRNA levels after RBP9 expression against the fold change after RBP10 expression (figure 2*c*). There is a correlation between the data (*R*^2^ = 0.33 at *t*_1_ and *R*^2^ = 0.58 at *t*_2_); the correlation is greater at *t*_2_ possibly because relatively few changes have occurred in the transcriptome of cells overexpressing RBP10 at *t*_1_. There is a clear trend for BSF transcript patterns as shown in figure 2*c*. Particular attention was paid to mRNAs that altered on expression of one RBP but not the other, a total of 269/1087 (approx. 24%) mRNAs uniquely regulated by RBP9 and 63/656 (approx. 9%) by RBP10 at *t*_2_. One such example is metacaspase MCA4 (Tb927.10.2440), and this observation was confirmed by northern blotting: MCA4 mRNA increased in response to RBP10 overexpression but not RBP9 (figure 2*d*). Two other metacaspases, MCA2 and MCA3, were also altered by RBP10 but not RBP9 (electronic supplementary material, table S1), and this might represent a specific RBP10-regulated family. Taken together, the RNA sequencing shows that either RBP10 or RBP9 overexpression is sufficient to confer a BSF-like transcriptome on PCFs.

### 3.4. Identification of putative RBP9 and RBP10 interacting proteins using BioID

Proximity-dependent BioID has been successfully applied to identify interacting proteins in several biological models including *T. brucei* [40,52–55]. The method is dependent on the expression of a transgene encoding a target protein-BirA* fusion that results in the biotinylation of proteins in close proximity to the target: the mean diffusion distance of the activated biotin is approximately 10 nm [54]. In these experiments, BirA*-RBP9 and BirA*-RBP10 were expressed from tetracycline-inducible transgenes and a BirA*-GFP transgene was used as a non-interacting control; the BirA*-GFP protein localized uniformly to the cytoplasm and nucleoplasm (not shown).

**Figure 3.**
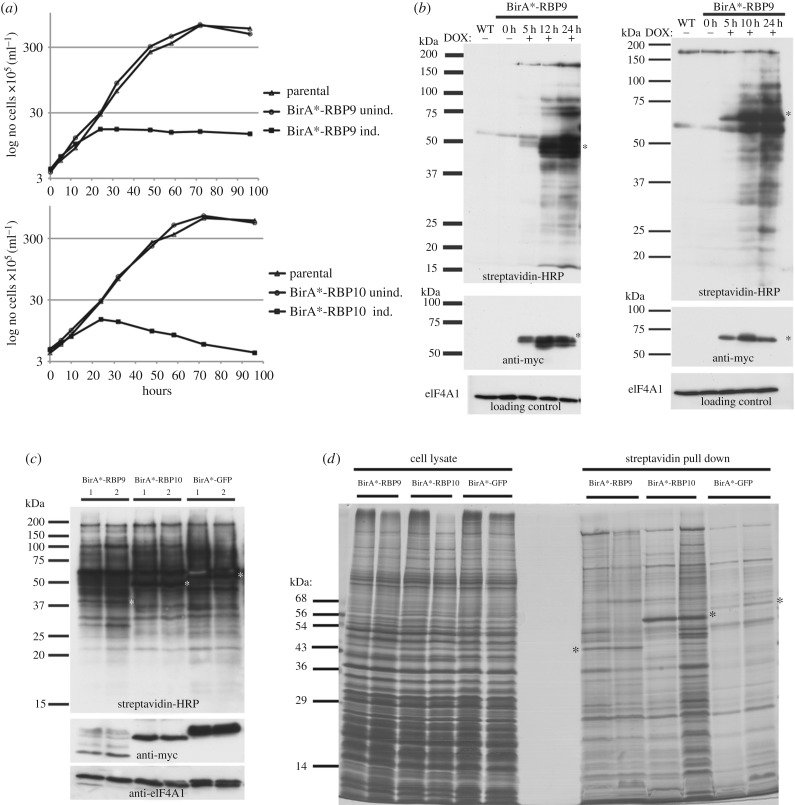
BirA*-RBP9 and BirA*-RBP10 biotinylated proteins. (*a*) Cumulative cell growth curve of cells lines showing the effect of overexpression of either BirA*-RBP9 (Lister 427 pSmOx p4522) or BirA*-RBP10 (Lister 427 pSmOx p4509). (*b*) *In vivo* biotinylation visualized by western blotting over a series of time points before and after induction of BirA*-RBPs. Streptavidin was used to detect biotinylation, anti-myc was used to detect induction of the transgenes and anti-eIF4A1 was loading control. (*c*) Western blot probed with streptavidin of the BirA*-RBP9, BirA*-RBP10 and BirA-GFP cell lysates used for subsequent pull downs 24 h post-induction (input). The white asterisks indicate the predicted weight of the BirA fusion proteins. Anti-myc was used to detect induction of the transgenes and anti-eIF2A was used as a loading control. (*d*) SDS-PAGE analysis of cell lystes and streptavidin pull downs. Each pull down was performed twice. The black asterisks mark BirA*-RBP9, BirA*-RBP10 and BirA*-GFP proteins.

Both the inducible BirA*-RBP9 and BirA*-RBP10 cell lines have the same proliferation phenotype described above for the wild-type and eYFP fusion proteins (figure 3*a*). To investigate whether biotinylation of nascent polypeptides occurred in actively translated mRNAs, the distribution of BirA*-RBP10 in fractions after sucrose gradient centrifugation of a cell lysate was determined 10 h post-induction. The BirA*-RBP10 was predominantly at the top of the gradient and a small amount was present in each fraction of the gradient including monosomes and polysomes, a similar distribution to BiP (electronic supplementary material, figure S5). This result was expected as the copy number of BirA*-RBP10 exceeds the total number of mRNA molecules per cell, let alone the fraction of mRNA molecules bound by RBP10. The experiment is inconclusive as it would not detect the interaction of a small fraction of BirA*-RBP10 with a subset of mRNAs. Next, BirA*-RBP9 and BirA*-RBP10 were induced and samples harvested over a time course to analyse biotinylation by western blotting with streptavidin. Complex patterns of biotinylation were apparent by 10–12 h (figure 3*b*), increasing to maximum levels after 24 h of transgene induction. Thus, the pull downs of biotinylated proteins were performed from cell lysates prepared after 24 h of transgene induction; at this point it is highly likely that many of the BirA*-RBPs were localized to P-bodies. Duplicate cell lysates were prepared and analysed for the level of biotinylation and transgene expression (figure 3*c*). All lysates contained a substantial number of biotinylated proteins despite having different levels of the BirA*-proteins: BirA*-GFP was expressed at higher levels than BirA*-RBP10 which in turn was expressed at higher levels that BirA*-RBP9. The biotinylated proteins purified using streptavidin beads contained both common and unique polypeptides for each of the fusion proteins and all patterns were different from the input lysate (figure 3*d*).

To identify proteins enriched in the RBP pull downs, proteins present in all six samples were identified by mass spectroscopy of tryptic peptides and a SINQ analysis [50] was performed comparing BirA*-RBP9 or BirA*-RBP10 with BirA*-GFP (electronic supplementary material, tables S5 and S6) [50]. In total, 772 and 782 proteins were present in both the replicates for BirA*-RBP9 and BirA*-RBP10 BioID pull downs, respectively. The BirA*-GFP pull down contained 1410 proteins present in both replicates (table 3; electronic supplementary material, tables S5–S8).

**Table 3.** BirA*-RBP9 and BirA*-RBP10 BioID-RNA-seq comparison at *t*_1_ and *t*_2_ time points. FC, fold change; FE, fold enriched.

	BirA*-RBP9				BirA*-RBP10			
	*t*_1_ (12 h)		*t*_2_ (24 h)		*t*_1_ (5 h)		*t*_2_ (10 h)	
	up	down	up	down	up	down	up	down
total	772				782			
total + ≥2FC RNA	26	80	39	146	9	13	25	59
total + ≥2FC RNA + dev. reg.	18	31	28	43	9	6	24	26
≥2×FE BioID	410				424			
≥2×FE BioID + ≥2FC RNA	21	45	26	73	9	5	20	35
≥2×FE BioID + ≥2FC RNA + dev. reg.	12	22	19	27	9	3	19	18
≥10×FE BioID	233				205			
≥10×FE BioID + ≥2FC RNA	15	40	17	44	7	4	12	17
≥10×FE BioID + ≥2FC RNA + dev. reg.	10	12	12	13	7	2	12	10

**Figure 4.**
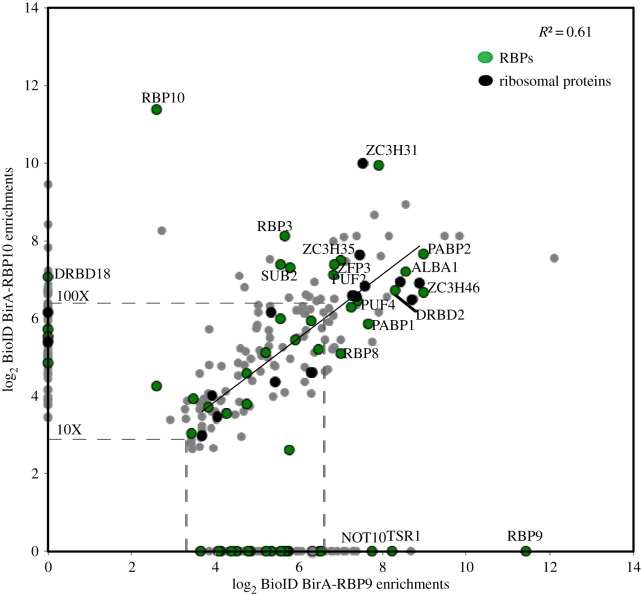
(*a*,*b*) Comparison of fold enrichments in biotinylation by BirA*-RBP9 and BirA*-RBP10 over a BirA*-GFP control. Green dots represent RNA-binding proteins, black dots ribosomal proteins and grey dots other proteins. Dashed lines indicate 10× and 100× fold enrichment.

Biotinylated proteins that were 10-fold enriched in BirA*-RBP9 and BirA*-RBP10 samples over the BirA*-GFP control values were considered for further analysis: this identified 233 proteins in BirA*-RBP9 and 205 in the BirA*-RBP10 pull downs. This analysis has some potential errors, for example an RBP interacting protein that was also a very abundant cytoplasmic protein may not be apparent. The datasets contained proteins that were present in one pull down but not the other and another set that was present in both (figure 4; electronic supplementary material, table S9). In all cases, there was over-representation of RNA-binding proteins. There were 81 proteins that were only present in the pull down from BirA*-RBP9 expressing cells (17 RBPs) and 59 proteins only present in the pull down from BirA*-RBP10 cells (4 RBPs), and 160 proteins in both (32 RBPs; figure 4; electronic supplementary material, table S10); 53/300 were RBPs. For proteins biotinylated by both BirA*-RBPs, the correlation between the enrichment in the two datasets was good (*R*^2^ = 0.61). This correlation means that some proteins were biotinylated at similar rates in the presence of the two different BirA*-RBPs.

Biotinylation of proteins by any BirA*-RBP can occur in a number of different situations. The biotinylated protein can be: (i) present in a complex with the BirA*-RBP bound to an mRNA; (ii) part of a complex that binds the same mRNA as, but is distinct from, the BirA*-RBP containing complex, for example a ribosome translating a mRNA that is also bound by BirA*-RBP; (iii) present in a small compartment with the BirA*-RBP separate from any mRNA; and (iv) the nascent polypeptide being synthesized from an mRNA that is bound by the BirA*-RBP. The possibilities are difficult to distinguish but there were many ribosomal proteins in the biotinylated fraction and it is probable that these were biotinylated while translating mRNAs bound by one or both RBPs. The BirA*-RBPs presumably localized to P-bodies like the eYFP-RBPs and it is likely that many of the proteins biotinylated by both BirA*-RBP9 and BirA*-RBP10 also co-localized to P-bodies. A comparison of the biotinylated proteins with the 232 proteins previously identified in a starvation granule enriched fraction [56] revealed substantial overlap, with 70/232 found in RBP9 BioID, 55/232 in RBP10 BioID and 49 present in both RBPs BioID (electronic supplementary material, table S11 and S12).

### 3.5. Proteins biotinylated by BirA*-RBP9

**Figure 5.**
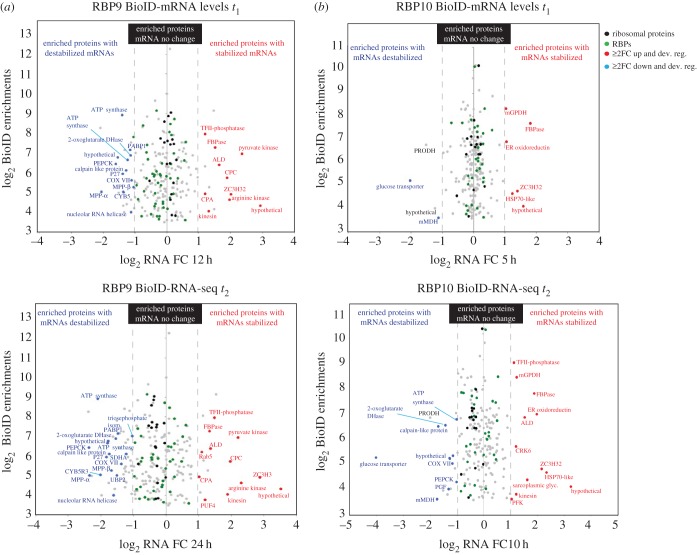
(*a*,*b*) Enrichment in biotinylation when compared with a BirA*-GFP control plotted against fold change (FC) in mRNA after induction of either a RBP9 (*a*) or a RBP10 (*b*) transgene at two time points after induction. Only those with a more than 10-fold enrichment in biotinylation are shown.

**Figure 6.**
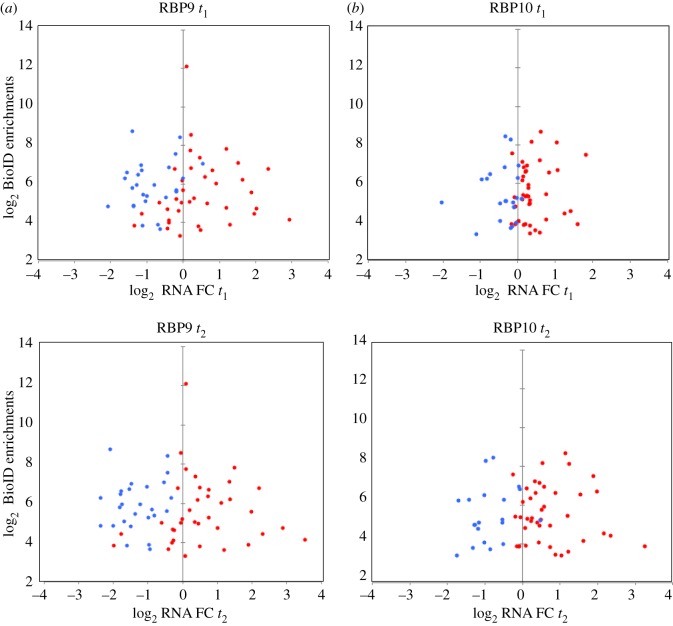
Enrichment in biotinylation when compared with a BirA*-GFP control plotted against fold change (FC) in mRNA after induction of either a RBP9 (*a*) or a RBP10 (*b*) transgene at two time points after induction for developmentally regulated mRNAs. Only those with a more than 10-fold enrichment in biotinylation are shown. Those normally upregulated in BSFs compared to PCFs are in red and those downregulated in blue.

For RBP9, 55/233 and 61/233 (*t*_1_ and *t*_2_, respectively) of the biotinylated proteins are encoded by mRNAs affected by RBP9 expression (figure 5*a*; electronic supplementary material, table S11). Of these, 22/55 and 25/61 (*t*_1_, *t*_2_) encoded developmentally regulated mRNAs stabilized by expression of RBP9. Indeed, when developmentally regulated mRNAs encoding proteins biotinylated by BirA*-RBP9 were plotted against fold change RNA values at *t*_1_ and *t*_2_, a pattern was found, with PCF mRNAs being downregulated and BSF mRNAs upregulated suggesting that many of these mRNAs may be directly bound by RBP9 (figure 6*a*).

**Figure 7.**
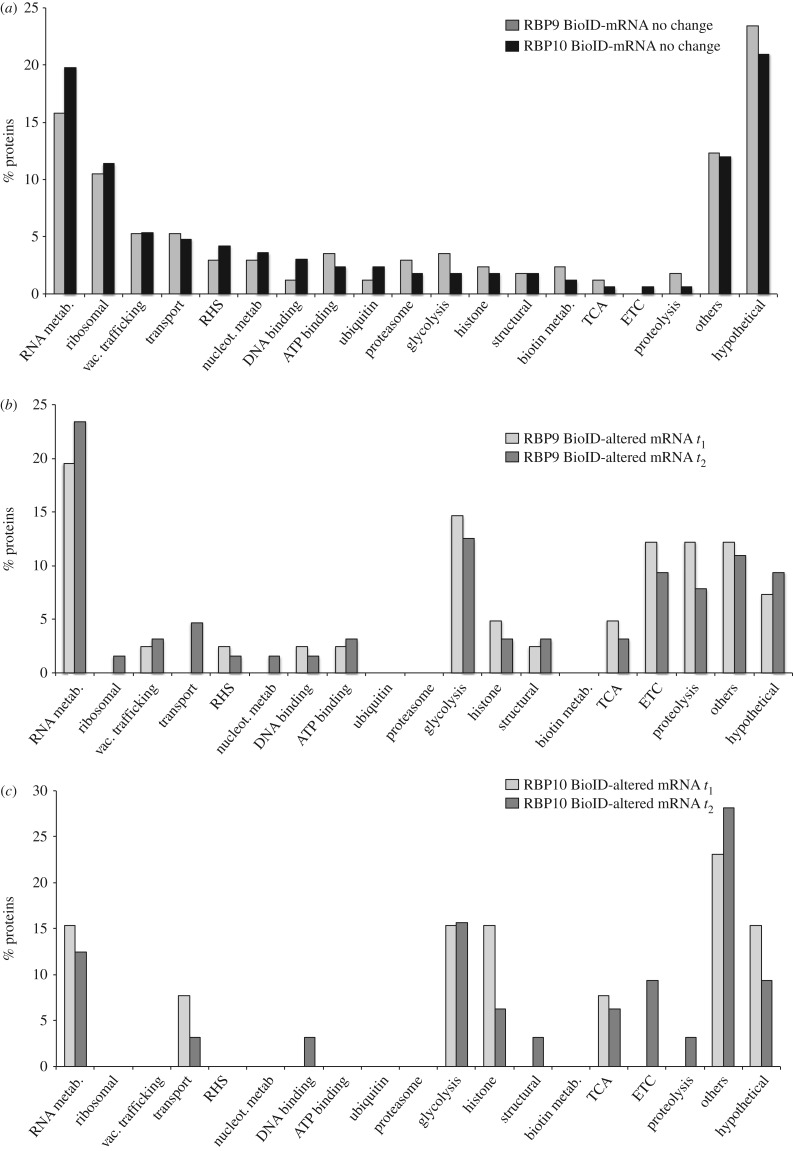
GO term analysis for proteins with more than 10-fold enrichment in biotinylation when compared with a BirA*-GFP control after induction of either a RBP9 or a RBP10 transgene. The categories were assigned based on GO terms or manually annotated and curated. RHS, retrotransposon hot spot proteins; TCA, tricarboxylic acid cycle; ETC, electron transport chain. (*a*) Biotinylated proteins encoded by an mRNA unaffected by RBP transgene induction, time point *t*_2_, *n* = 167 for RBP9 and *n* = 171 for RBP10. (*b*). Biotinylated proteins encoded by an mRNA altered by RBP9 transgene induction. Light grey, *t*_1_ (*n* = 41); dark grey, *t*_2_ (*n* = 64) *(c*). Biotinylated proteins encoded by an mRNA altered by RBP10 transgene induction. Light grey, *t*_1_ (*n* = 13); dark grey, *t*_2_ (*n* = 32).

To investigate any functional differences between proteins identified by RBP9 BioID with and without changes in their mRNA levels, the two groups of proteins were classified into families based on GO terms and manual annotations (figure 7; electronic supplementary material, table S11). For those that were biotinylated and encoded by mRNAs unaffected by RBP9 overexpression, ribosomal proteins and proteins related to RNA metabolism were over-represented (GO:0003735, structural constituent of ribosome (*p* = 2.7 × 10^−7^); GO:0003723: RNA binding (*p* = 1.3 × 10^−5^); figure 7), confirming the successful enrichment of components related to the mRNA metabolism of the cell. Of the total number of proteins associated with RNA metabolism, 13/43 RBPs were also regulated at the RNA level at *t*_2_; the remaining 30/43 RBPs were probably in proximity to RBP9 in the cell but could also be encoded by mRNAs unaffected by RBP9 binding. Excluding hypothetical proteins and proteins classified as ‘others’, the next most enriched type of proteins is ‘associated with vacuolar trafficking and transport’.

A similar analysis for biotinylated proteins encoded by mRNAs regulated by RBP9 overexpression revealed that several categories were greatly reduced when compared with the above (figure 7*b*; electronic supplementary material, table S11). There was almost no representation of vacuolar trafficking, transport, ribosomal proteins, ubiquitin or the proteasome (figure 7*b*; electronic supplementary material, table S11). By contrast, families related to energy metabolism, many aspects of which are developmentally regulated (electron transport chain, glycosome–glycolysis or tricarboxylic acid cycle) together with the term ‘proteolysis’ increased significantly its percentage and numbers (figure 7*b*; electronic supplementary material, table S11 and figure S6).

### 3.6. Proteins biotinylated by birA*-RBP10

For RBP10, 11/205 and 29/205 (*t*_1_, *t*_2_, respectively) of the proteins identified by BioID were encoded by mRNAs affected by overexpression of RBP10 (figure 5*b*; electronic supplementary material, table S12). Of these, 9/11 and 22/29 (*t*_1_, *t*_2_) were also developmentally regulated mRNAs, indicating the selective effect of RBP10 overexpression. The enrichment of biotinylated proteins was plotted against their fold change RNA values at *t*_1_ and *t*_2_ (figure 5*b*): this showed the presence of BSF upregulated proteins in both biotinylated and mRNA regulated fractions, suggesting a direct interaction of RBP10 with these mRNAs. The analysis of functional differences between the proteins identified by RBP10 BioID without a change in mRNA and those that also altered mRNA levels showed again that ribosomal proteins and proteins related to RNA metabolism were overrepresented in the first category (GO:0003735, structural constituent of ribosome (*p* = 1.29 × 10^−6^); GO:0003723: RNA binding (*p* = 3.8 × 10^−3^); figure 7*a*; electronic supplementary material, table S11). Of the proteins associated with RNA metabolism, only 2/31 RBPs were also regulated at the RNA level at *t*_2_; this is a smaller fraction than for RBP9. Furthermore, 6/31 RBPs were also developmentally regulated RBPs. In the fraction of proteins identified by RBP10 BioID that were encoded by mRNAs affected by RBP10 overexpression, categories involved in basic energetic metabolism such as electron transport chain, glycosome glycolysis or tricarboxylic acid cycle were significantly increased (figure 7*c*; electronic supplementary material, table S12 and figure S6), again suggesting developmental regulation.

### 3.7. Comparison of RBP9 with RBP10

The RBP9 and RBP10 datasets were compared to identify any differences. For those proteins identified by BioID and encoded by mRNAs regulated by RBP overexpression, differences between the total number of proteins for BirA*-RBP9 and BirA*-RBP10 (61 versus 29 at *t*_2_ in 10-fold or greater enriched fraction) was attributed to the larger number of mRNAs that change when RBP9 was induced (table 3). A greater fraction linked to ‘proteolysis’ (GO: 0006508) was identified for RBP9 (*p*-value = 0.039; 7/232) compared to RBP10 (3/202). For RBP9, this included two downregulated mitochondrial peptidases (Tb927.11.3980 and Tb927.5.1060) and three upregulated cathepsins (Tb927.6.1030, Tb927.6.1000, Tb927.6.560) that are all developmentally regulated (figure 7*b*,*c*). Among the proteins identified by BioID without any change in the cognate mRNAs, the RNA helicase DHH1 was 3.3- and 4.3-fold enriched in RBP9 and RBP10, respectively; DHH1 is known to affect the developmental regulation of some mRNAs [26]. SCD6, a protein that binds DHH1, was 3.05- and 3.8-fold enriched in RBP9 and RBP10, respectively (electronic supplementary material, figure S7). This level of enrichment can be interpreted in two ways: (i) DHH1 and SCD6 might localize in the proximity of RBP9 and RBP10, or be very transiently associated; and (ii) DHH1 and SCD6 are interactors with the RBPs but the degree of enrichment after BioID is low as they are both very abundant proteins and so are represented in the non-specific BioID pool.

## 4. Discussion

Very little is known about the composition and dynamics of mRNPs implicated in developmental transitions in mRNA abundances in trypanosomes. RBP9 and RBP10 were selected for this study as they are encoded by the two RBP mRNAs that show the greatest differential upregulation in BSFs compared with PCFs [23,57], and for RBP9 mRNA also when compared to epimastigotes [57]. RBP10 overexpression in PCFs has been shown to be sufficient to induce movement towards a BSF-like transcriptome [32]. When tethered to a reporter mRNA in PCFs, both RBP9 and RBP10 caused translational repression [58].

In this work, RBP9 and RBP10 were individually overexpressed to investigate their function. First, expression of either causes a proliferation arrest. The effect on proliferation is rapid, detectable within 12 h for RPB9 and 5 h for RBP10; this effect is independent of the presence of an N-terminal tag, eYFP or BirA*. Both eYFP-tagged RBPs co-localize with DHH1 and SCD6, which are markers for P-bodies, and this co-localization was most apparent as the P-body number per cell increases as proliferation stops. Second, overexpression of either RBP9 or RBP10 resulted in changes in the transcriptome that were similar and many of the changes mimicked the pattern of mRNAs in BSFs. However, there was a small subset of mRNAs that responded to overexpression of one RBP but not the other: this suggests that there were some targets unique to each RBP and that the two RBPs are not operating solely as ordered components in a cascade. In an attempt to distinguish primary effects of RBP overexpression from secondary effects, changes in the transcriptome were analysed at timepoints before proliferation arrest (*t*_1_): 12 h for RBP9 and 5 h for RBP10.

At *t*_1_, a large percentage of the affected transcripts were normally developmentally regulated (366/709 for RBP9 and 175/261 for RBP10) and many of these were related to central metabolism, see above (table 2). The experiments investigating the effect of RBP9 or RBP10 overexpression in PCFs showed that they could individually push the pattern of mRNA expression towards a BSF pattern. This is not too surprising as both RBPs are normally expressed in BSFs and it has previously been shown that RBP6, inducibly expressed in PCFs, allows these cells to continue with their developmental cycle [28]. This progression through developmental stages is unlikely to occur in the experiments described here, as the effect of RBP9 and RBP10 is to force the developmental cycle into reverse.

Why are two distinct RBPs each able to produce a BSF-like mRNA expression pattern? It is possible that both are needed or that one is necessary for the epimastigote to metacyclic transition and the other for the metacyclic to BSF transition.

The interactions of the two RBPs was investigated using inducible expression of each RBP with an N-terminal BirA* tag. This leads to proximity-dependent biotinylation of surrounding molecules and preferential biotinylation of interacting proteins. Inducible expression of GFP with an N-terminal BirA* tag was used as a control, on the assumption that it has no specific interactors and would biotinylate cytoplasmic and nuclear proteins in proportion to their abundance. The BirA*-RBP fusions clearly biotinylated proteins in a proximity-dependent manner as both were the most abundantly biotinylated proteins in their own datasets. Other specifically biotinylated proteins were identified by enrichment over the control and this identified sets of proteins, some of which overlapped whereas others were distinct. The largest category of proteins identified for both RBPs was ‘RNA metabolism’: primarily other RBPs and ribosomal proteins. This proximity to a number of RBPs is consistent with a localization to cytoplasmic P-bodies, which are known to contain a large number of RNA metabolism proteins, especially when cell proliferation slows or stops [56]. The biotinylation of ribosomal proteins from both the large and small subunit could well indicate that the RBPs are associated with mRNAs being actively translated. The polysomal profiling of BirA*-RBP10 was inconclusive and it remains possible that a small fraction of BirA*-RBP10 is polysome-associated although the vast majority is not after over-expression.

On comparing the RNA-seq and BioID data, a significant proportion of proteins identified by BioID were also regulated at the mRNA level after RBP9 (55/232 (*t*_1_) and 61/232 (*t*_2_)) and RBP10 (11/202 (*t*_1_) and 29/202 (*t*_2_)) induction (figure 5). Many of these mRNAs are normally developmentally regulated and this is reflected in an enrichment in GO terms for central metabolism (figure 6 and table 3; electronic supplementary material, table S3) as PCFs use amino acids as a carbon source and oxidative phosphorylation to generate ATP and reducing equivalents, whereas BSFs use glycolysis. The biotinylation of these proteins could result from proximity of the nascent polypeptide to the BirA*-RBP bound to its cognate mRNA as opposed to a direct interaction with the RBP.

How far did these BioID experiments go in further characterizing the RBPs? mRNAs that were altered by RBP expression and also encoded proteins that were biotinylated are likely to interact with the RBP. Otherwise, it is more difficult to make a link between biotinylation and function. The level of enrichment of ribosomal proteins in the biotinylated fraction suggests that both RBPs were associated with actively translated mRNAs. However, this simple interpretation is complicated by the localization of the RBPs to P-bodies; this means it is not possible to distinguish between proteins biotinylated as part of mRNP particles in the cytoplasm and proteins that co-localize to P-bodies. There is a substantial overlap between proteins present in the starvation granule-enriched fraction [56] and the proteins identified by BioID. A further difficulty in obtaining readily interpretable data is the low activity of the BirA* domain as even with high levels of overexpression it took hours to achieve a degree of labelling sufficient for analysis.

In conclusion, a combination of BioID with RNA-seq has been used to dissect mRNP composition, but the data also highlight the complexity of the life cycle of an mRNP.

## Supplementary Material

Table S1

## Supplementary Material

Table S2

## Supplementary Material

Table S3

## Supplementary Material

Table S4

## Supplementary Material

Table S5

## Supplementary Material

Table S6

## Supplementary Material

Table S7

## Supplementary Material

Table S8

## Supplementary Material

Table S9

## Supplementary Material

Table S10

## Supplementary Material

Table S11

## Supplementary Material

Table S12

## Supplementary Material

Figure S1-S7

## Data accessibility

The raw reads obtained from the ectopic expression in PCFs of eYFP-RBP9 and eYFP-RBP10 (*t*_1_ and *t*_2_) and non-induced cell lines (*t*_0_) have been deposited in the ENA (European Nucleotide Archive) under the accession number PRJEB14125 (http://www.ebi.ac.uk/ena/data/view/PRJEB14125). The full set of raw reads from each biological replicate of Lister 427 PCF and BSF form RNA was deposited to EBI array express under the accession number E-MTAB-3335. All the datasets supporting this article have also been uploaded as part of the electronic supplementary material.
