# Supplementary material for: Characterization of RBP9 and RBP10, two developmentally regulated RNA-binding proteins in Trypanosoma brucei
Source: Open Biol. 2017 Apr 5;7(4):160159. doi: 10.1098/rsob.160159 (PMC5413900; doi:10.1098/rsob.160159)
Supplement: Figure S1-S7 [file rsob160159supp13.pdf]

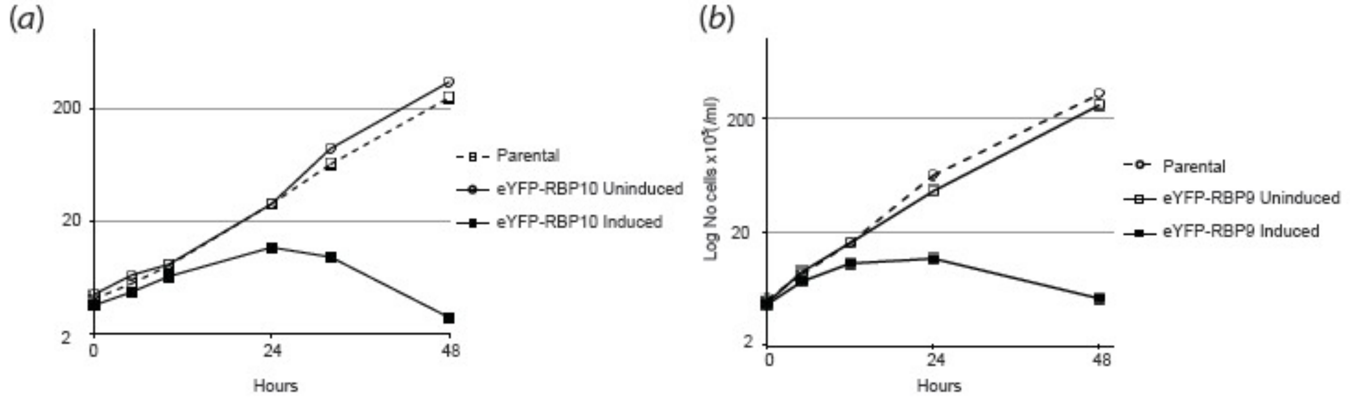


**Figure S1.** Growth curve of parental, Induced (+Dox) and uninduced(-Dox) for eYFP-RBP9 (*a*) and eYFP-RBP10 (*b*).

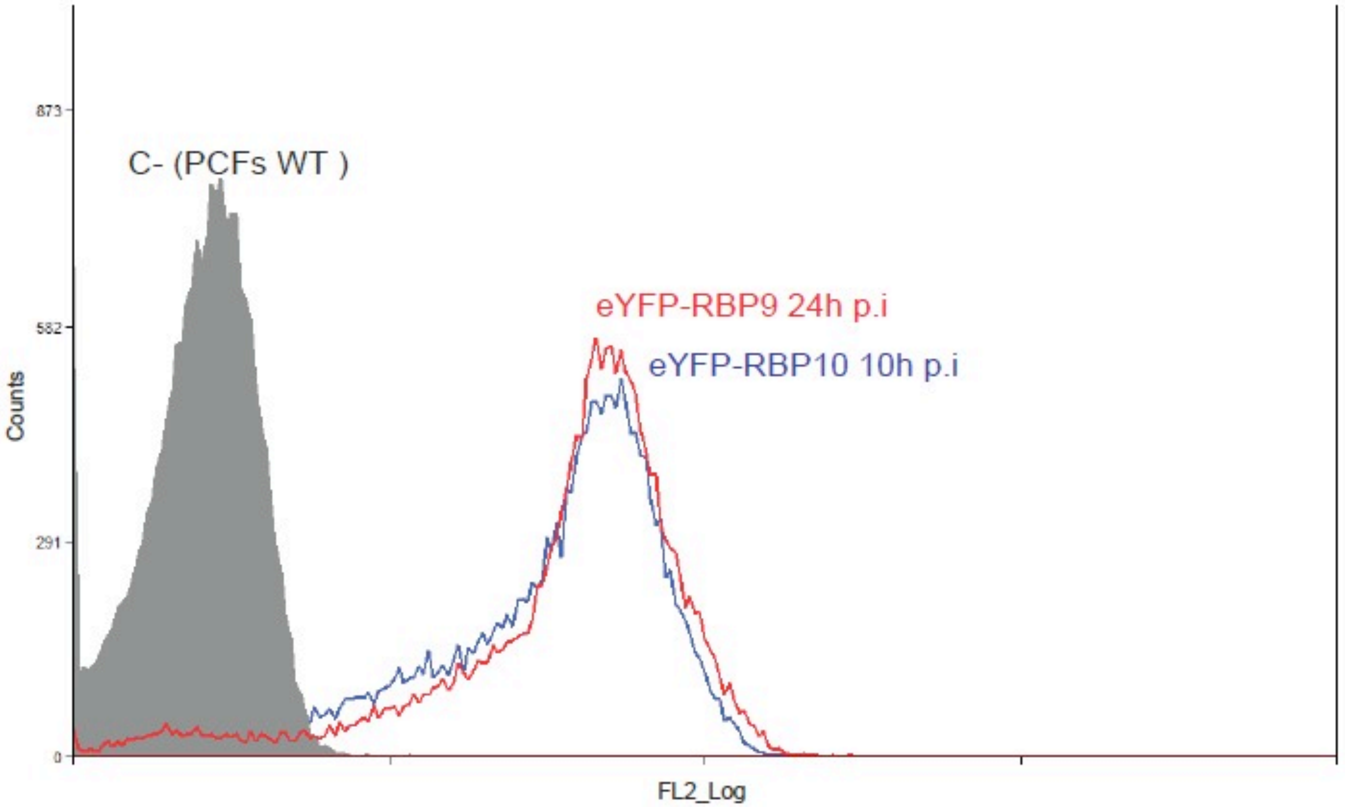


**Figure S2.** FACS analysis for the eYFP expression of the non-induced (C-), eYFP-RBP9 24 hours post-induction (t2) and eYFP-RBP10 10 hours postinduction (t2) cell lines

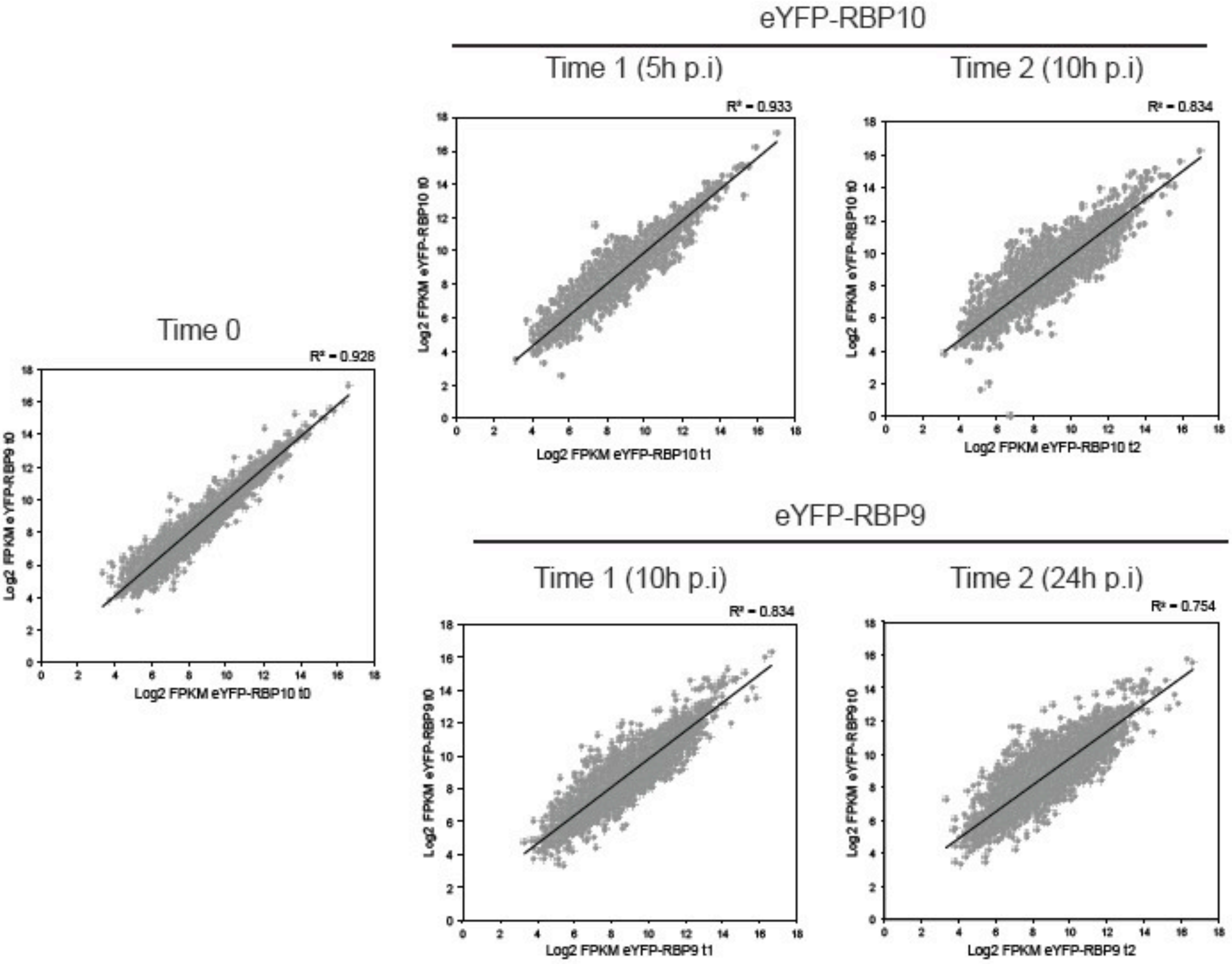


**Figure S3.** *Trypanosoma brucei* procyclic RNA-seq comparisons at t0, t1 and t2 post-induction of eYFP-RBP10 and eYFP-RBP9 proteins.

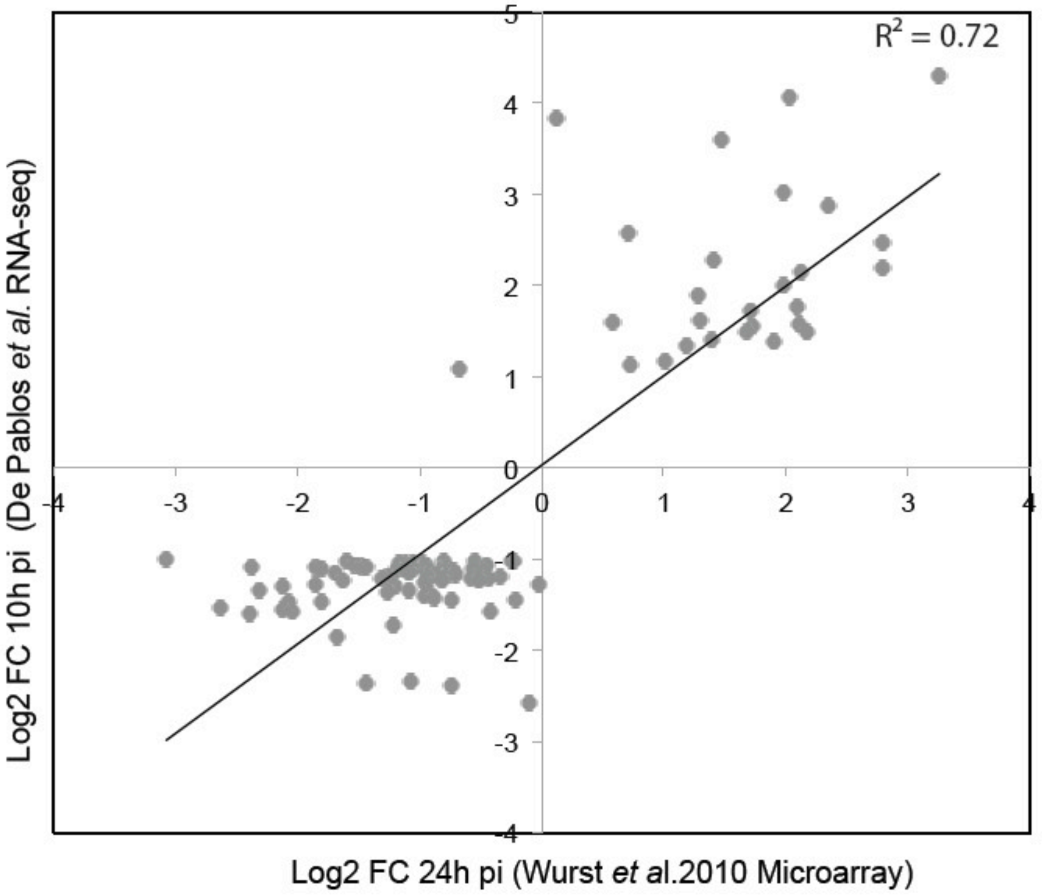


**Figure S4.** Scatter plot comparison of ≥ 2 FC Up-regulated and Down- regulated RNAs after RBP10 overexpression in PCFs. Log2 FC 10 h pi RNA- seq in the y-axis and Log2 FC 24 h pi microarray data from Wurst et al., 2010. n=99 genes (25 Up-regulated, 74 Down-regulated).

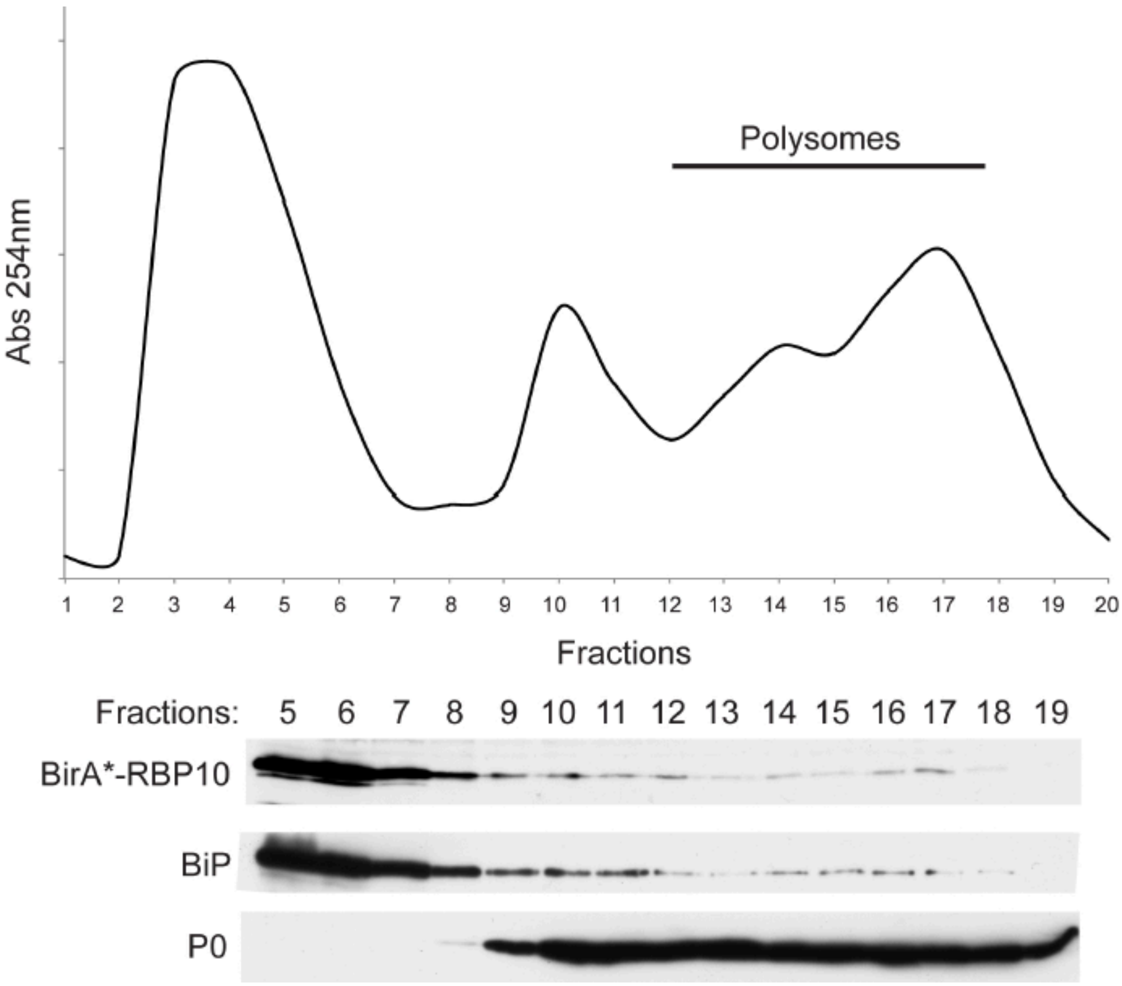


Figure S5. Distribution of BirA*-RBP10 in sucrose density gradient fractions. Lysates of cell lines expressing the transgene for 10 h were fractionated by sucrose gradient centrifugation, and the absorption profiles at 254nm as well as western blotting using anti-myc antibodies were used to analyze the fractionation of BirA*-RBP10. BiP protein was used as negative control of polysomal location and Ribosomal P0 as control of ribosomal protein presence in the different fractions.

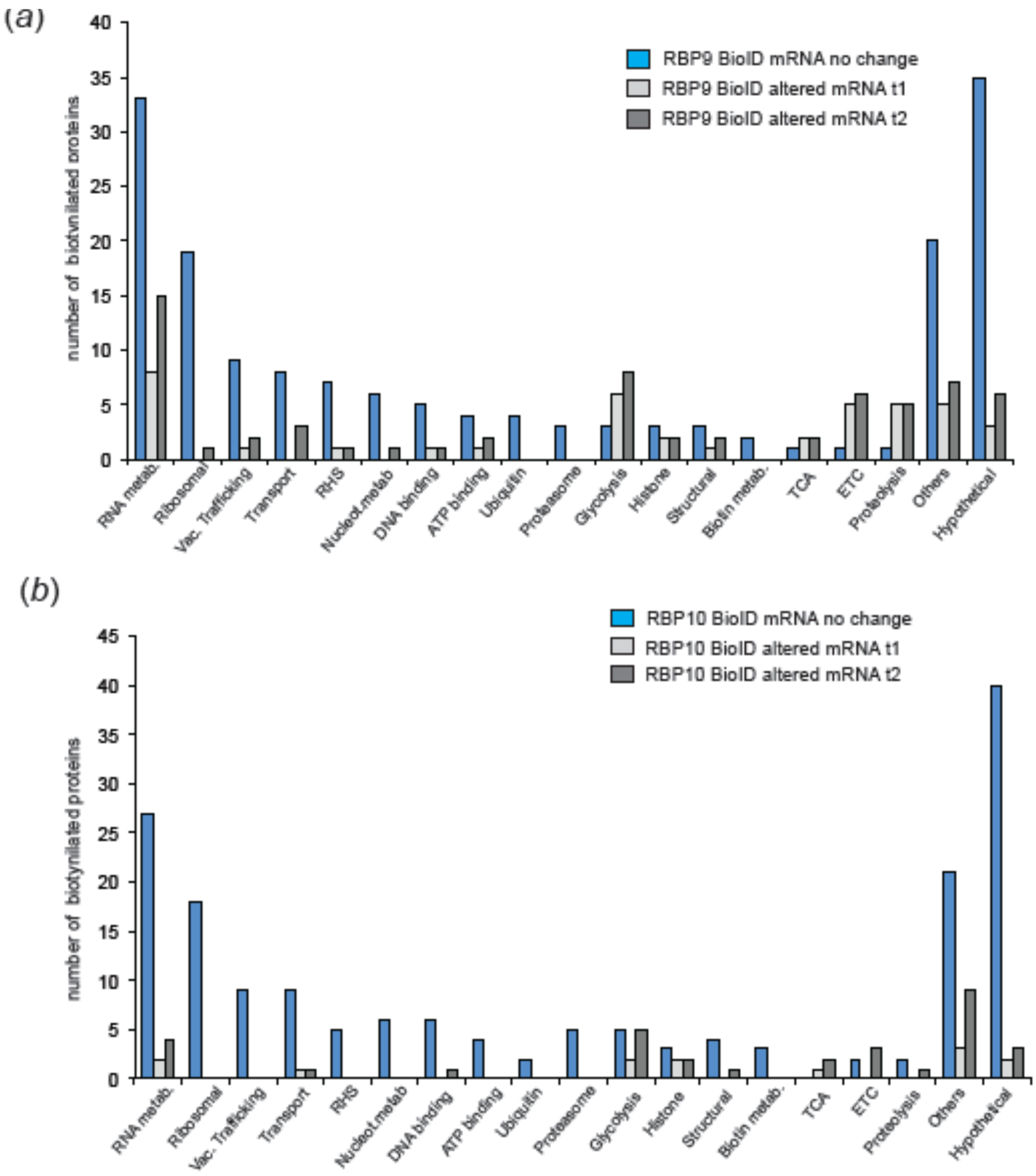


**Figure S5.** Representation of the proteins present in each protein group in the ≥10 Fold enriched fraction for the interactor and nascent peptide categories. (*a*). RBP9 biotinylated proteins present in the "Interactor" category at t2 are represented by Blue bars (n=167), light and dark grey bars represent the proteins falling within the "nascent peptide" category (n=41 and n=64, respectively). (*b*). RBP10 biotinylated proteins present in the "Interactor" category at t2 are represented by blue bars (n=171), light and dark grey bars represent the proteins falling within the "nascent peptide" category (n=13 and n=32, respectively). The categories were assigned based on GO terms or manually annotated and curated. Abbreviations: RHS: Retrotransposon Hot Spot Proteins; TCA: Tricarboxylic acid cycle; ETC: Electron transport chain

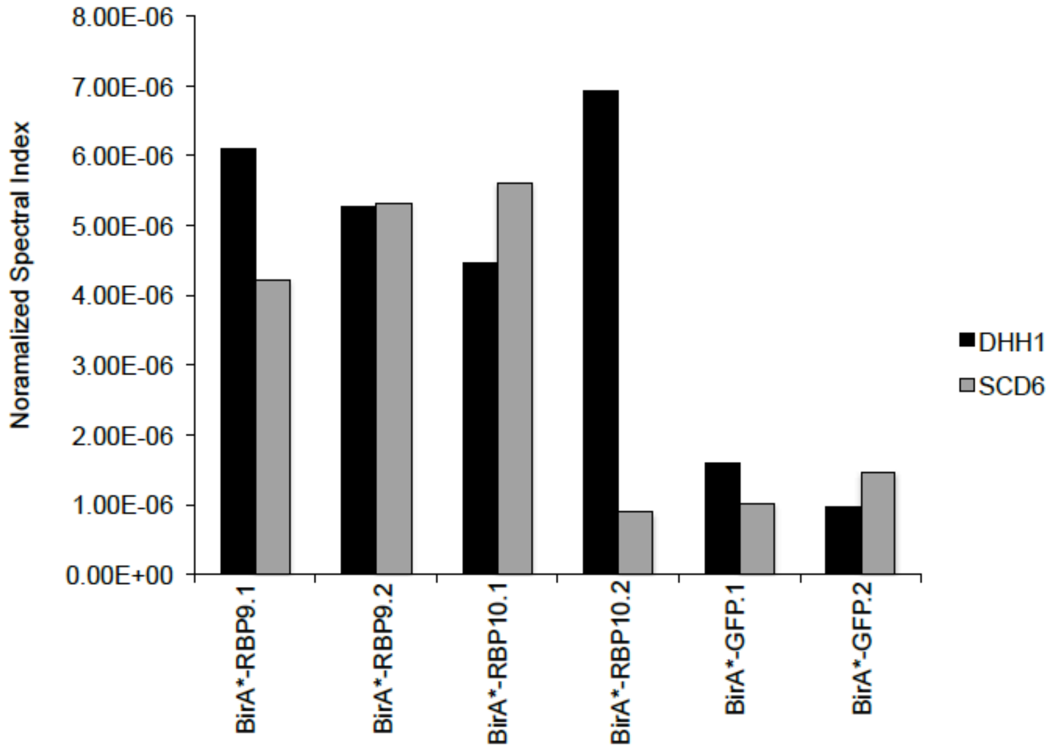


**Figure S6.** Normalized Spectral Index of DHH1 and SCD6 proteins after induction of BirA*-RBP9, BirA*-RBP10 or BirA*-GFP.
